# Feasibility of a prospective real-time monitoring system capturing real-world data from elderly patients with acute myeloid leukemia

**DOI:** 10.1016/j.esmorw.2026.100778

**Published:** 2026-09-22

**Authors:** S.J.F. Hermans, J. Versluis, S. Langhout, E.D. van Werkhoven, G. Huls, D. Chitu, P.E. Westerweel, R.B. Fiets, L.W. Tick, A.H.E. Herbers, M.F. Corsten, D.A. Breems, I.S. Nijhof, M.J. Cruijsen, Y. Sandberg, M. Davidis, A. Koster, K.G. van der Hem, L. van der Burg, F. Baboe, E.F.M. Posthuma, K. Schade, E. Berkx, A.G. Dinmohamed, P.C. Huijgens, J.J. Cornelissen

**Affiliations:** 1 Department of Hematology, Erasmus MC Cancer Institute, University Medical Center Rotterdam, Rotterdam, The Netherlands; 2 Performation Healthcare Intelligence, Zeist, The Netherlands; 3 HOVON Foundation, Rotterdam, The Netherlands; 4 Department of Hematology, University Medical Center Groningen (UMCG), Groningen, The Netherlands; 5 Department of Hematology, Albert Schweitzer Hospital, Dordrecht, The Netherlands; 6 Department of Hematology, Amphia Ziekenhuis, Breda, The Netherlands; 7 Department of Hematology, Maxima Medical Center, Veldhoven, The Netherlands; 8 Department of Hematology, Jeroen Bosch Hospital, Den Bosch, The Netherlands; 9 Department of Hematology, Meander Medisch Centrum, Amersfoort, The Netherlands; 10 Department of Hematology, ZNA Stuivenberg/Middelheim, Antwerp, Belgium; 11 Department of Hematology, Sint Antonius Ziekenhuis, Nieuwegein, The Netherlands; 12 Department of Haematology-Oncology, Catherina Hospital, Eindhoven, The Netherlands; 13 Department of Internal Medicine, Maasstad Hospital, Rotterdam, The Netherlands; 14 Department of Hematology, Beatrix Hospital, Gorinchem, The Netherlands; 15 Department of Hematology, VieCuri Hospital, Venlo, The Netherlands; 16 Department of Hematology, Zaans Medical Center, Zaandam, The Netherlands; 17 Department of Internal Medicine, Antonius Hospital, Sneek, The Netherlands; 18 Department of Internal Medicine, Bravis Hospital, Roosendaal, The Netherlands; 19 Department of Hematology, Reinier de Graaf Gasthuis, Delft, The Netherlands; 20 Department of Research and Development, Netherlands Comprehensive Cancer Organisation (IKNL), Utrecht, The Netherlands

**Keywords:** acute myeloid leukemia, external comparator, external control arm, external control cohort, randomized controlled trial, real-world data

## Abstract

**Background:**

Real-world data (RWD) are increasingly applied as external controls in phase II–III clinical trials. However, retrospectively monitored RWD may suffer from limitations, including data availability and quality. Prospective monitoring of RWD may potentially overcome such limitations. We addressed the feasibility of an automated monitoring system for prospectively capturing RWD of older patients with acute myeloid leukemia (AML) and compared them with patients in the control arm of the HOVON-155 (H155) AML trial.

**Materials and methods:**

The system extracted predefined data from patients receiving nonintensive therapy in 10 Dutch hospitals. Patients in the RWD cohort were further selected for comparison with patients in the H155-trial by applying the trial eligibility criteria. Caliper propensity score matching (PSM) of patients in the RWD cohort with patients in the H155-control arm was performed based on age, European LeukemiaNet 2017, and Hematopoietic Cell Transplant-Comorbidity Index.

**Results:**

Between 2020 and 2025, 193 patients in the RWD cohort were identified, of whom 120 had not participated in the H155-trial but did meet its eligibility criteria. Patients in the H155 control arm (*n* = 70) and patients in the RWD cohort (*n* = 120) differed in baseline parameters. Using PSM, 51 patients in the RWD cohort were compared with 51 patients in the H155-control arm. The 100-day mortality proportion rate was 37.3% versus 27.5% (*P* = 0.40) among patients in the H155 control arm and patients in the RWD cohort, respectively. Two-year overall survival was 18% ± 5% versus 18% ± 5% (hazard ratio 1.05, 95% confidence interval 0.69-1.62, *P* = 0.81). Bleeding and bacteremia appeared to occur more frequently among patients in the H155 control arm, whereas the frequency of other adverse events was comparable.

**Conclusion:**

A prospective RWD monitoring system was efficiently applied in 10 Dutch hospitals. Although patient characteristics initially differed from the H155 control arm, the outcome of the RWD cohort appeared similar following PSM. Prospective monitoring of RWD seems feasible but matching is needed for future use in clinical studies.

## Highlights

• RWD are increasingly applied as external control cohorts to single-arm trials.
• Comparisons using retrospective RWD may be hampered methodologically and statistically.
• A prospective RWD monitoring system was developed and validated in 10 Dutch hospitals.
• Those RWD showed similar outcomes as the control arm of a contemporaneous randomized trial.
• The monitoring system is a feasible approach to overcome some limitations of retrospective RWD.

## Introduction

Real-world data (RWD) have extensively been incorporated into postmarketing safety monitoring by regulatory agencies and cost-effectiveness assessments by Health Technology Assessment bodies.^1^^,^^2^ Relatively recently, RWD have been applied as an external control cohort to supplement or replace the control arm of clinical trials, such as phase II single-arm trials.^3^, ^4^, ^5^ A recent systematic review of hemato-oncology trials by our group, however, identified that the use of RWD as external control arms may introduce several methodological and statistical limitations, which challenge the reported findings.^6^ Limitations included the lack of comorbidity data to fully allow for adherence to trial inclusion and exclusion criteria, differences in endpoint definitions, and insufficient reporting of toxicities. Furthermore, bias due to the retrospective nature of most RWD further hampered comparability, such as by changes in standard care over time. To potentially overcome the limitations of retrospectively monitored RWD, an automated digital data pipeline to monitor RWD prospectively was developed and validated.^7^ It was reported that this prospective monitoring system captured diagnosis data of (hemato-)oncology patients in accordance with the eligibility criteria of The Netherlands Cancer Registry (NCR) and it was also able to extract follow-up data, such as treatment regimens and toxicities. In this study, we addressed the feasibility of that system to extract data from older patients with acute myeloid leukemia (AML) for potential use as an external control cohort and evaluated their comparability to the control arm of the recently completed HOVON-155 (H155) trial.^8^ This randomized phase II trial included older patients with AML or high-risk myelodysplastic syndrome (MDS) who received decitabine with or without midostaurin.^8^ According to the target trial framework,^9^ feasibility was defined by the effective generation of a contemporaneous cohort of patients with AML or high-risk MDS that would meet the H155 trial eligibility criteria and emulate similar characteristics and outcomes as the control arm of the H155 trial.

## Methods

### Patient eligibility criteria

Recently, a prospective monitoring system (‘Datagateway’) was developed and validated to supplement the NCR with prospectively monitored data, both at diagnosis and in follow-up, thus creating a single comprehensive dataset of Datagateway and NCR data, herein referred to as ‘RWD cohort’.^7^ The system applied a combination of International Statistical Classification of Diseases and Related Health Problems (10th Revision) codes and treatment data, which was tailored in this analysis to identify all newly diagnosed, consecutive cases of AML or high-risk MDS in older adults in 10 Dutch hospitals. In addition, the system captured treatment, outcome, and toxicity data. This approach yielded a cohort of patients for whom data were automatically extracted. As a second step, the available H155 trial eligibility criteria were applied to that cohort. Adult patients with AML or high-risk MDS were eligible for inclusion in the external control cohort if (i) they were newly diagnosed since 1 January 2020, (ii) had a WHO performance status (WHO-PS) ≤2 at diagnosis (or unknown WHO-PS), (iii) were not previously included in HOVON-SAKK trials, (iv) received hypomethylating agents without additional drugs, and (v) had complete survival data. As a result, 120 out of 193 (62.2%) initially identified RWD patients were included in the comparison with the H155 control arm (Supplementary Figure S1, available at https://doi.org/10.1016/j.esmorw.2026.100778). A more detailed description of the H155 clinical trial and the data monitoring system that yielded patients in the RWD cohort is provided in the Supplementary Methods (available at https://doi.org/10.1016/j.esmorw.2026.100778).

### Statistical analysis

The primary endpoint of this analysis was overall survival (OS) measured from the date of randomization in the H155 trial and the start of treatment in the RWD cohort. OS was estimated using Kaplan–Meier analysis and Cox proportional hazards models. Equivalence with respect to OS using a 10% margin was investigated by the two one-sided tests (TOST) procedure. The median follow-up time was estimated using the inverse Kaplan–Meier method. Early mortality was compared based on the proportion of patients who died within the first 60 and 100 days after diagnosis, respectively. The available data elements were harmonized to improve comparability of both patient populations (Supplementary Methods and Table S1, available at https://doi.org/10.1016/j.esmorw.2026.100778). An overview of the statistical software is given in the Supplementary Material (available at https://doi.org/10.1016/j.esmorw.2026.100778).

### Harmonization of RWD and trial control patients

Patient cohorts were also harmonized to improve similarity, which was carried out in a three-step model. Firstly, patient data were obtained using the automated monitoring system. Secondly, RWD patients were considered for inclusion by manual application of H155 trial eligibility criteria. Thirdly, a 1 : 1 nearest-neighbor caliper propensity score matching (PSM) algorithm was applied to improve comparability of baseline characteristics of the external RWD cohort with H155 control arm patients,^10^, ^11^, ^12^ which included age, European LeukemiaNet 2017 (ELN2017),^13^ and hematopoietic cell transplantation (HCT)-specific comorbidity index (HCT-CI).^14^ Patients without available ELN2017 were classified separately. A caliper width of 0.2 standard deviation of the propensity score was applied in accordance with guidance by Austin.^15^ Sensitivity analyses, as well as the balance assessments before and after matching, are detailed in the Supplementary Material (available at https://doi.org/10.1016/j.esmorw.2026.100778).

## Results

### Patient characteristics

The patient selection process included three steps: automated extraction, manual selection based on H155 trial eligibility criteria, and PSM. As a first step, the automated system identified 193 RWD patients with AML or high-risk MDS who were newly diagnosed between 2020 and 2025. Of these 193 RWD patients, 120 met the available eligibility criteria of the H155 trial (second step) and were eligible for comparison with the H155 control arm (*n* = 70). Significant differences in baseline characteristics emerged, including age and white blood cell count (Table 1). To optimize comparability (third step), RWD patients were 1 : 1 matched to the H155 control arm patients, using age, ELN2017 risk, and HCT-CI, which resulted in 51 RWD patients being matched to 51 patients of the H155 control arm (Table 2, Supplementary Table S2 and Figure S2, available at https://doi.org/10.1016/j.esmorw.2026.100778). At diagnosis, patients in the H155 control arm (*n* = 51) had a median age of 74 years (range 57-88) and patients in the RWD cohort (*n* = 51) had a median age of 77 years (range 38-88, *P* = 0.10; Table 2). Patients in the H155 control arm were numerically more often male (66.7% versus 60.8%, *P* = 0.68) and were more frequently diagnosed with AML (82.4% versus 68.6%, *P* = 0.17). Among patients in the RWD cohort, 73.3% had a WHO-PS of 0 or 1, compared with 86.3% of patients in the H155 control arm (*P* = 0.31). Furthermore, WHO-PS was not recorded for 21 patients in the RWD cohort. An ELN2017 adverse risk was observed in the majority of patients in both cohorts. Most patients in both cohorts had one or more comorbidities, as measured by the HCT-CI. The median white blood cell count was higher among patients in the H155 control arm than among patients in the RWD cohort (3.9 versus 2.8 × 10^9^/l, *P* = 0.03). The majority of patients in the RWD cohort (92.2%) received azacitidine, whereas all patients in the H155 control arm received decitabine according to the study protocol.

**Table 1:** Patient characteristics of H155 control arm patients and RWD patients in the unmatched cohorts

Parameter	H155 control arm (*n* = 70)	RWD (*n* = 120)	*P* value
Age at diagnosis, median (range)	74 (57-88)	77 (38-90)	0.01
Male sex, *n* (%)	47 (67.1)	67 (55.8)	0.17
Diagnosis year, median (range)	2021 (2020-2021)	2022 (2020-2025)	
Diagnosis,^a^*n* (%)			0.06
AML	58 (82.9)	84 (70.0)	
High-risk MDS	12 (17.1)	36 (30.0)	
Trial participation, *n* (%)	70 (100)	0	
WHO performance status, *n* (%)			0.41
0	22 (31.4)	20 (35.1)	
1	36 (51.4)	23 (40.3)	
2	12 (17.2)	14 (24.6)	
Unknown	0	63	
ELN 2017 risk classification, *n* (%)			0.14
Favorable	5 (8.5)	3 (7.5)	
Intermediate	19 (32.2)	6 (15.0)	
Adverse	35 (59.3)	31 (77.5)	
Unknown	11	80	
HCT-CI, *n* (%)			0.09
0	12 (17.1)	29 (24.1)	
1-2	19 (27.1)	44 (36.7)	
≥3	39 (55.8)	47 (39.2)	
White blood cell count (10^9^/l), median (range)	3.9 (0.7-28.4)	2.9 (1.0-9.4)	0.04
Missing	0	0	
Treatment regimen, *n* (%)			
Azacitidine	–	111 (92.5)	
Azacitidine and decitabine	–	4 (3.3)	
Decitabine	70 (100)	5 (4.2)	
Missing	–	–	
AlloSCT, *n* (%)	4 (5.7)	4 (3.3)	0.47
Median follow-up, months (95% CI)	44 (34-NR)	36 (26-46)	

alloSCT, allogeneic stem cell transplant; AML, acute myeloid leukemia; CI, confidence interval; ELN, European Leukemia Net; H155, HOVON-155; HCT-CI, hematopoietic cell transplantation-specific comorbidity index; ICD, International Classification of Diseases and Related Health Problems; MDS, myelodysplastic syndrome; NR, not reached; RWD, real-world data; WHO, World Health Organization.

a Diagnoses in the RWD cohort were derived using ICD-10 classifications.

**Table 2:** Patient characteristics of H155 control arm patients and RWD patients in the matched cohorts

Parameter	H155 control arm (*n* = 51)	RWD (*n* = 51)	*P* value
Age at diagnosis, median (range)	74 (57-88)	77 (38-88)	0.10
Male sex, *n* (%)	34 (66.7)	31 (60.8)	0.68
Diagnosis year, median (range)	2020 (2020-2021)	2022 (2020-2025)	
Diagnosis,^a^*n* (%)			0.17
AML	42 (82.4)	35 (68.6)	
High-risk MDS	9 (17.6)	16 (31.4)	
Trial participation, *n* (%)	51 (100)	0	
WHO performance status, *n* (%)			0.31
0	17 (33.4)	10 (33.3)	
1	27 (52.9)	12 (40.0)	
2	7 (13.7)	8 (26.7)	
Unknown	0	21	
ELN 2017 risk classification, *n* (%)			1.00
Favorable	4 (10.0)	3 (7.7)	
Intermediate	7 (17.5)	6 (15.4)	
Adverse	29 (72.5)	30 (76.9)	
Unknown	11	12	
HCT-CI, *n* (%)			1.00
0	10 (19.6)	9 (17.7)	
1-2	14 (27.5)	15 (29.4)	
≥3	27 (52.9)	27 (52.9)	
White blood cell count (10^9^/l), median (range)	3.9 (0.7-28.1)	2.8 (1.2-9.4)	0.03
Missing	0	0	
Treatment regimen, *n* (%)			
Azacitidine	–	47 (92.2)	
Azacitidine and decitabine	–	2 (3.9)	
Decitabine	51 (100)	2 (3.9)	
Missing	–	–	
AlloSCT, *n* (%)	3 (5.9)	1 (2.0)	0.62
Median follow-up, months (95% CI)	44 (26-NR)	36 (22-NR)	

alloSCT, allogeneic stem cell transplant; AML, acute myeloid leukemia; CI, confidence interval; H155, HOVON-155; HCT-CI, hematopoietic cell transplantation-specific comorbidity index; ICD, International Classification of Diseases and Related Health Problems; MDS, myelodysplastic syndrome; NR, not reached; RWD, real-world data; WHO, World Health Organization.

a Diagnoses in the RWD cohort were derived using ICD-10 classifications.

### OS

Median follow-up times were 44 months (95% confidence interval [CI]: 26-not reached [NR]) and 36 (95% CI: 22-NR) for the matched H155 control arm patients and RWD cohorts, respectively (Table 2). We evaluated early mortality and OS in the matched cohorts (Table 3). In the H155 control arm, 13 patients (25.5%) died within the first 60 days compared with 11 patients (21.6%) in the RWD cohort (*P* = 0.82). The 100-day mortality was 37.3% (*n* = 19) among patients in the H155 control arm versus 27.5% (*n* = 14) among patients in the RWD cohort (*P* = 0.40). OS at 2 years [estimate ± standard error (SE)] was 18% ± 5% among patients in the H155 control arm versus 18% ± 5% among matched patients in the RWD cohort [hazard ratio (HR) = 1.05, 95% CI 0.69-1.62, *P* = 0.81; Figure 1]. Coarsened exact matching (CEM), exact matching (EM) followed by caliper PSM, and inverse probability of treatment weighting (IPTW) were applied to evaluate the impact of the choice of matching algorithm on the OS estimates. All three sensitivity analyses demonstrated comparable OS estimates and HRs (Supplementary Table S3, available at https://doi.org/10.1016/j.esmorw.2026.100778). Equivalence for OS at 2 years was tested via the TOST procedure using a 10% margin. The nonrounded OS difference was 0.50% between cohorts (SE for the difference 0.076%, 95% CI ranging from −14.5% to 15.5%). TOST resulted in a *P* value of 0.11. As this study involved a nonrandomized comparison, unmeasured confounders may have influenced the observed OS estimates. Previously, Vanderweele and Ding^16^ introduced the concept of an ‘*E* value’ to determine the minimum HR that an unmeasured confounder needs to exert in order to move the observed HR into the direction of 1.0. An *E* value of 1.22 was observed for the HR estimate for OS, with an *E* value of 1.0 for the lower bound of the 95% CI.

**Table 3:** Outcome of H155 control arm patients and RWD patients in the matched cohorts

Parameter	H155 control arm (*n* = 51)	RWD (*n* = 51)	*P* value
60-day mortality,^a^*n* (%)	13 (25.5)	11 (21.6)	0.82
100-day mortality,^a^*n* (%)	19 (37.3)	14 (27.5)	0.40
2-year OS^b^ (estimate ± SE)	18 ± 5	18 ± 5	0.81

H155, HOVON-155; OS, overall survival; PSM, propensity score matching; RWD, real-world data; SE, standard error.

a 60-day and 100-day mortality were calculated as the proportion of patients who died within 60 or 100 days since the start of treatment, without accounting for censoring.

b OS estimates at 2 years after randomization or diagnosis in the unmatched cohorts were 23% ± 5% versus 23% ± 4% (*P* = 0.74) for patients in the H155 control arm (*n* = 70) and patients in the RWD cohort (*n* = 120), respectively.

**Figure 1:**
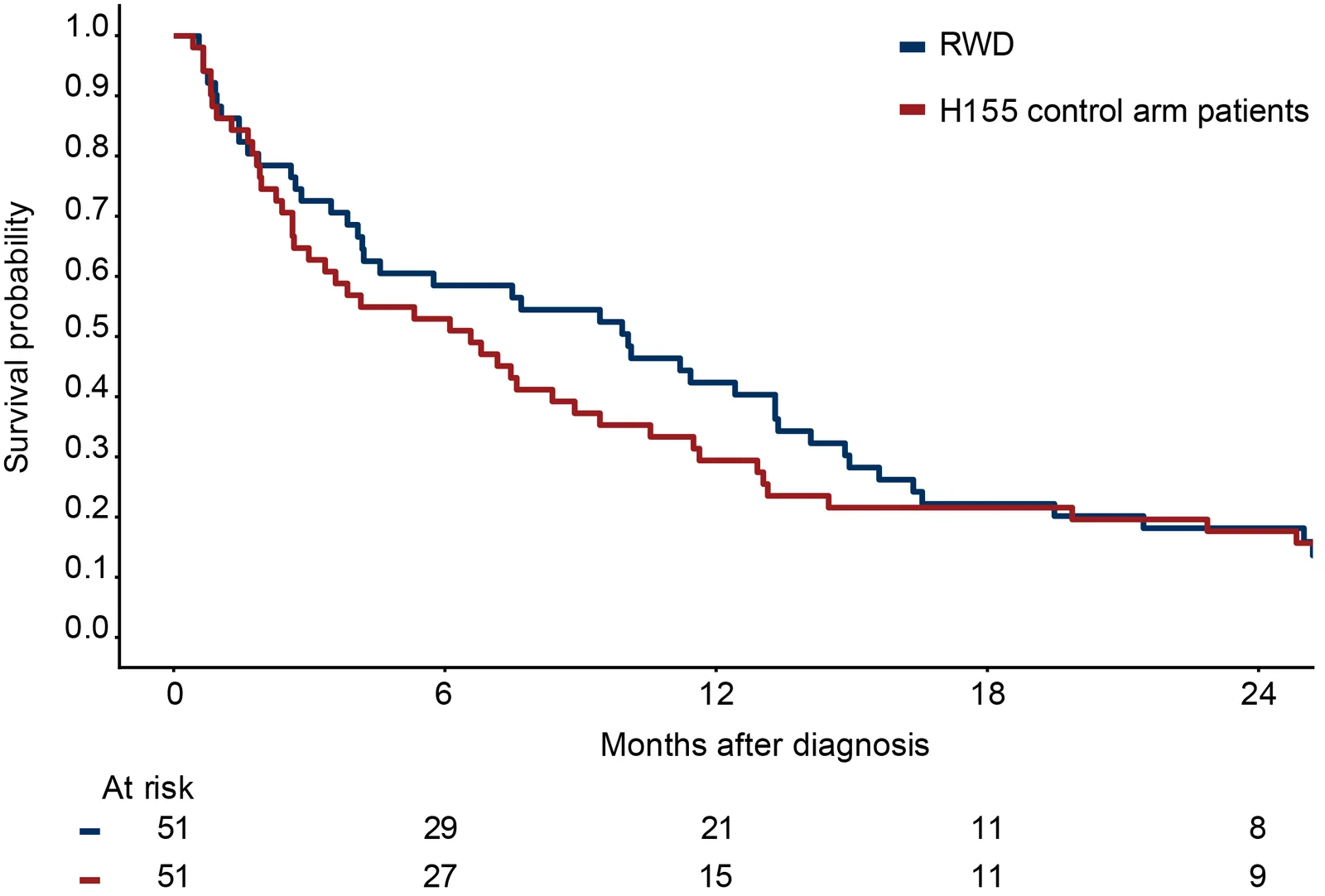
**Overall survival for H155 control arm patients versus RWD patients in the matched cohorts.** This figure details the OS among patients in the H155 control arm and RWD cohort after 1 : 1 nearest-neighbor caliper propensity score matching for age, ELN2017 AML risk classification, and HCT-CI score. AML, acute myeloid leukemia; ELN, European LeukemiaNet; H155, HOVON-155; HCT-CI, hematopoietic cell transplantation–specific comorbidity index; OS, overall survival; RWD, real-world data.

### Toxicities

Adverse events [Common Terminology Criteria for Adverse Events (CTCAE) 5.0 grade ≥3] were routinely monitored in the H155 trial and were extracted in accordance with CTCAE 5.0 within the first 6 months after start of treatment for the RWD cohort, as defined and reported previously.^7^ Subsequently, the occurrence of the first adverse event (per category) was compared for both cohorts. The incidences of acute renal insufficiency, intensive care unit admission, hepatic toxicity, thrombosis, and pulmonary aspergillosis are detailed in Table 4 and were rather comparable between matched H155 control arm patients and RWD patients. However, grade ≥3 bleeding and bacteremia were more frequently observed among patients in the H155 control arm than among those in the RWD cohort, occurring in seven versus three patients (13.7% versus 5.9%) and four versus two patients (7.8% versus 3.9%), respectively. The incidence of adverse events in the unmatched cohorts is detailed in Supplementary Table S4 (available at https://doi.org/10.1016/j.esmorw.2026.100778).

**Table 4:** Reported toxicity (CTCAE grade ≥3) of H155 control arm patients and RWD patients in the matched cohorts

Parameter	H155 control arm (*n* = 51)	RWD (*n* = 51)
Acute renal insufficiency grade ≥3, *n* (%)	2 (3.9)	2 (3.9)
Bleeding grade ≥3, *n* (%)	7 (13.7)	3 (5.9)
Intensive care unit admission, *n* (%)	–	–
Hepatic toxicity grade ≥3, *n* (%)	2 (3.9)	3 (5.9)
Bacteremia, *n* (%)	4 (7.8)	2 (3.9)
Venous thrombosis grade ≥3, *n* (%)	–	2 (3.9)
Pulmonary aspergillosis, *n* (%)	4 (7.8)	4 (7.8)

CTCAE, Common Terminology Criteria for Adverse Events; H155, HOVON-155; RWD, real-world data.

### Comparing external RWD with the H155 experimental arm for OS

Lastly, we investigated the feasibility of using the RWD cohort as supplementary external controls by comparing OS between patients in the H155 experimental arm (*n* = 70) and those in the H155 control arm (*n* = 70), and between patients in the H155 experimental arm and the RWD patients identified after matching cohort (*n* = 51; Figure 2A and B). In the original trial, estimated OS at 2 years was 23% ± 5% among patients in the H155 control arm versus 13% ± 4% among patients in the H155 experimental arm (*P* = 0.27; Figure 2A).^8^ Estimated OS at 2 years was 18% ± 5% among the matched RWD patients cohort versus 13% ± 4% among patients in the H155 experimental arm (HR 0.91, 95% CI 0.62-1.33, *P* = 0.63; Figure 2B).

**Figure 2:**
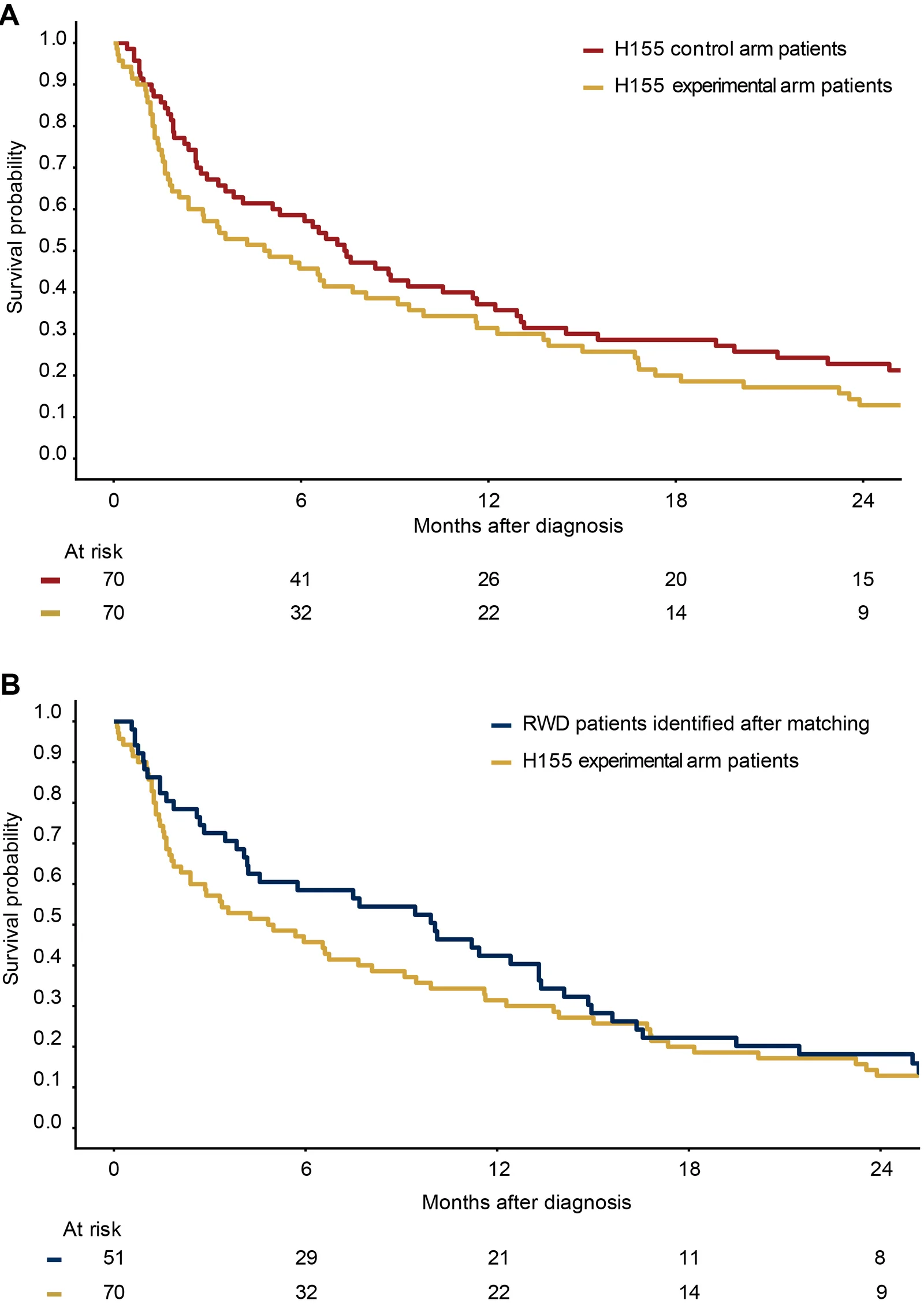
**OS among patients in the H155 experimental arm under various scenarios.** This figure illustrates the comparison of OS among patients in the H155 experimental arm versus patients in the H155 control arm and matched RWD patients. (A) The red and yellow curves illustrate the OS of the H155 control and experimental arms as reported in the original publication, respectively. (B) The blue curve and the yellow curve illustrate the scenario in which patients in the RWD cohort who were identified after matching (*n* = 51) were used as the control arm for the H155 experimental arm (red curve, *n* = 70). H155, HOVON-155; OS, overall survival; RWD, real-world data.

## Discussion

Although randomized controlled trials (RCTs) are the gold standard to evaluate experimental treatment modalities in hematology-oncology, targeted treatment in tightly defined subgroups of patients hampers the design and completion of large RCTs. Therefore, alternative designs are currently being explored to address these shortcomings. RWD are more frequently considered as external control data in nonrandomized studies, especially in the field of (hemato-)oncology.^4^^,^^5^^,^^17^ However, quality of data and bias due to retrospective monitoring of RWD are important limitations.^17^, ^18^, ^19^, ^20^ We developed a prospective monitoring system to identify RWD patients who might be eligible for prospective clinical trials in AML. More specifically, in this study, we focused on older patients with AML or high-risk MDS in the RWD cohort and compared them with patients in the control arm of the randomized phase II clinical H155 trial, which enrolled older patients with AML or high-risk MDS who received HMAs.^7^^,^^8^ A total of 193 concurrent RWD patients were identified, including 120 patients who met the available trial eligibility criteria. Subsequently, these patients were matched with patients in the H155 control arm based on age, ELN2017 AML risk, and comorbidities. It resulted in comparable baseline characteristics, similar 2-year OS, and largely similar incidences of grade ≥3 toxicities. Although the 2-year point estimate suggests similar outcomes, we cannot exclude a clinically relevant difference of 10% or more based on equivalence testing. Altogether, the approach shows the feasibility of such a prospective RWD monitoring system that meets some of the most important limitations associated with retrospective monitoring of RWD.^6^

Several previously identified key issues in the application of external (real-world) control data need to be considered,^6^ such as nonconcurrent inclusion periods, incomplete application of trial eligibility criteria to the RWD cohort, dissimilarity in endpoints between the trial arm and external control cohort, and incomplete reporting of toxicity data. Additional factors of importance are included the geographic origin of patients in the RWD and missing data within cohorts. Regarding inclusion periods, patients in the H155 control arm were diagnosed and enrolled between January 2020 and October 2021. To improve comparability of the RWD cohort with the H155 control arm, only patients in the RWD cohort who were diagnosed from 2020 onward were considered. The upper limit of the inclusion period was not restricted to 2021 to identify as RWD patients as possible within the 10 hospitals across The Netherlands that implemented the monitoring system. Furthermore, the inclusion and exclusion criteria of the H155 trial were applied to the RWD cohort to the extent available in the registry data. In contrast to most RWD studies,^6^ exclusion criteria were also considered by incorporating comorbidity data in the RWD cohort. In this study, patients in the RWD cohort were also matched with patients in the H155 control arm based on HCT-CI. Although this is not a replacement for the strict application of exclusion criteria, it might offer an improvement compared with disregarding exclusion criteria altogether, because organ function (e.g. heart ejection fraction or pulmonary function tests) and (geriatric) comorbidity assessments, here by means of HCT-CI, are important prognostic parameters in older patients with AML.^21^, ^22^, ^23^, ^24^, ^25^, ^26^, ^27^, ^28^ Missing data in the RWD cohort most often occurred due to clinicians not actively denoting data in the patients’ records and these could therefore not be supplemented. In general, RWD registries lack toxicity data, which require an intensive monitoring system using clearly defined toxicities based on existing grading systems. In this study, toxicity data were included in the prospective monitoring system, allowing comparison of several clinically relevant adverse events between patients in the H155 control arm and those in the RWD cohort. We previously validated treatment toxicities in patients with AML who received intensive and nonintensive treatment, with varying accuracy.^7^ In this study, it was observed that the frequencies of adverse events were similar between cohorts, except for bleeding and bacteremia. These events might be underrepresented in the RWD as a consequence of pragmatic query definitions. Grade ≥3 bleeding events in the RWD cohort were defined based on radiology or gastrointestinal endoscopy findings of bleeding, whereas common other bleeding sources (e.g. urinary tract or epistaxis) were not routinely captured. This was similarly observed in our validation study with an accuracy of bleeding adverse events of 87%.^7^ Similarly, grade ≥3 bacteremia is defined by CTCAE as positive blood cultures with signs or symptoms for which treatment is indicated, whereas the prospective monitoring system captured patients with positive blood cultures who subsequently received antibiotics. It suggests that the prospective monitoring system was less able to identify these bleeding and bacteremia events as compared with the manual reporting within a prospective RCT but testing in a larger sample size is required to verify if there is a real difference.

Although the automated system efficiently captured the most important data, some limitations remained. Similarity of endpoint definition in the trial and external cohorts is required to allow for a valid comparison.^29^ The primary endpoint of this analysis was OS, which was reliably captured in the system. However, treatment response (i.e. rate of complete remission with or without incomplete recovery) or event-free survival (EFS) were included as primary and secondary endpoints in the H155 trial, respectively, for which data could not be prospectively monitored reliably. A feedback loop from the automated signaling system notified NCR data registrars if a bone marrow biopsy was carried out in follow-up. Registrars intended to manually collect response data for up to four cycles of chemotherapy in the RWD cohort but these data were not systematically recorded by treating physicians outside a clinical trial setting. As a result, it precluded the inclusion of response rate and EFS in this analysis.

The outcome of the RWD cohort was better than the outcome of the experimental treatment arm of the H155 trial, similar to the original control arm. It might be explained by a higher incidence of relapse and relapse mortality in patients who received decitabine and midostaurin, as suggested in the original report.^8^ It would have been informative to determine whether the incidence of relapse in the RWD cohort was similar to the incidence observed among patients in the H155 control arm. Unfortunately, it was not possible to include relapse and cause of death in the automated system as they were stored in the hospital patient records in formats inaccessible to the monitoring system.

Automated extraction systems have also been applied by others but, to our knowledge, not in the context of a clinical trial.^30^, ^31^, ^32^ For example, Abler et al.^30^ evaluated a semiautomated pipeline to extract response data from positron emission tomography–computed tomography imaging and estimated progression-free survival that was similar to the reference data. Similarly, automatically extracted data on patients with breast cancer, including PDF documents by the use of text mining, were combined into a single repository for monitoring care pathways.^31^ Lastly, chemotherapy data were extracted from a nationwide prescription platform and deemed comparable to hospital source data.^32^ Despite different use cases, these experiences may still inform the analysis presented here. A future application of similar text mining tools might allow not only for reporting toxicities but also treatment response and relapse data. Including these data might enable to optimize the comparison of RWD control cohorts with trial internal control patients for endpoints such as treatment response and EFS.^33^

Altogether, a prospective monitoring system that adhered to the trial case report form was implemented to identify patients in the RWD cohort who might serve as potential external controls for the randomized phase II H155 trial in older patients with AML or high-risk MDS who received nonintensive treatment. Matched RWD patients cohort demonstrated similar baseline characteristics and outcomes compared with patients in the H155 control arm. Some limitations associated with retrospective monitoring of RWD were overcome.^6^^,^^17^ These results suggest that the capturing system is feasible and may be applied on a broader scale.
