# Supplementary material for: Feasibility of a prospective real-time monitoring system capturing real-world data from elderly patients with acute myeloid leukemia
Source: ESMO Real World Data Digit Oncol. 2026 Sep 22;14:100778. doi: 10.1016/j.esmorw.2026.100778 (PMC13634072; doi:10.1016/j.esmorw.2026.100778)
Supplement: Supplementary file 1 [file mmc1.pdf]

Supplementary Appendix

This is the appendix to Hermans et al. - Feasibility of a prospective real-time monitoring system capturing Real-World Data from elderly patients with acute myeloid leukemia

Table of contents

Supplementary Methods 3

Supplementary Table 1. Availability and definition of data elements in the HOVON and RWD cohorts 6

Supplementary Table 2. Assessment of the matching procedures 7

Supplementary Table 3. Survival outcome of H155 control arm patients and RWD patients under various matching algorithms 8

Supplementary Table 4. Reported toxicity (CTCAE grade ⩾3) of H155 control arm patients and RWD patients in the unmatched cohorts 9

Supplementary Figure 1. CONSORT diagram for inclusion of H155 control arm patients and Real-World Data (RWD) patients 10

Supplementary Figure 2. Distribution of propensity scores for H155 control arm patients and RWD patients in the unmatched and matched cohorts 11

References 12

Supplementary Material

Supplementary Methods

HOVON-155 trial design

The H155 trial was a randomized phase II clinical trial that evaluated the addition of midostaurin to decitabine in older AML patients who were either unfit for intensive chemotherapy, or elected non-intensive treatment.[1] These drugs were evaluated in an octopus design. Patients were randomized from January 2020 till October 2021, and were eligible for inclusion if (1) a diagnosis of AML or high-risk (Revised International Prognostic Scoring System ≥ 4.5) myelodysplastic syndrome (MDS) was newly established; (2) patients had a WHO performance status (WHO-PS) ≤2 at diagnosis (or unknown WHO-PS). Patients in the control arm (H155 control arm patients; n=70) received 5 to 10-day courses of decitabine (20 mg/m2) until progression of disease was observed, after which they continued with 5-day cycles. In the present analysis, we included all eligible H155 control arm patients for comparison with the Real-World Data (RWD) cohort, but excluded all experimental arm patients (Supplementary Figure 1).

Prospectively monitored RWD patients

The Netherlands Cancer Registry (NCR) is a national cancer registry, maintained by the Netherlands Comprehensive Cancer Organization, in which 95% of all newly diagnosed cancer patients in the Netherlands are retrospectively recorded.[2] Originally, the NCR registered cancer diagnoses by combining data from the Netherlands Histopathology Registry (“PALGA”) and financial claims data from Dutch Hospital Data, supplemented with retrospective patient chart review. The RWD cohort was identified using an automated data extraction system that is currently implemented in ten Dutch hospitals. The technical details of the validation of that system are reported in a paper by our group.[3] The automated system extracted data from electronic health records using prespecified and validated queries from both structured and unstructured fields by using MySQL, and free-text search strings for the

extraction of adverse events. These queries are based on a standardized data dictionary (e.g., Data S1 and Tables S1, S2 and S6 in Langhout et al.[3]). Currently, the system does not incorporate informatics methods such as natural language processing. Extracted data were sent to the NCR. Simultaneously, data registrars of the NCR extracted data elements from electronic health records, as is routinely performed for virtually all cancer patients in the Netherlands. The dataset used in this analysis contained all automated data elements, and the data elements entered by the registrars, each in separate variables. Data elements were combined when necessary, such as to update the overall survival status.

Harmonization of baseline and outcome parameters

Although the RWD cohort was prospectively monitored, harmonization of some baseline and outcome parameters was needed as the definition and availability differed between the cohorts (Supplementary Table 1). Regarding the European LeukemiaNet 2017 (ELN2017) risk classification,[4] an intermediate risk was assigned for RWD patients in whom no favorable or adverse risk aberrations were observed. In addition, patients with inconclusive cytogenetic or molecular data were classified as “unknown” risk.

Sensitivity analyses and estimation of residual confounding

To evaluate the potential effect of the choice of matching algorithm, sensitivity analyses were performed using exact matching (EM), coarsened exact matching (CEM), and EM followed by propensity score matching (PSM).[5] Lastly, we also applied inverse probability of treatment weighting (IPTW). Histograms that detailed the distribution of the propensity scores in the H155 control arm patients and the RWD cohort were used to visually inspect the matching analysis, while standardized mean differences and variance ratios were used to more formally assess the matching procedure. We applied E-values to quantify the required strength of an unobserved confounder to nullify the observed OS estimate, as has been previously recommended in the context of non-randomized studies.[6] This E-value is

expressed on the risk ratio scale by default but can be transformed to a hazard ratio scale, and is as such reported here.[6]

Statistical software

Data management was performed using STATA (STATACorp 2019. Stata Statistical Software: Release 18. College Station, TX: StataCorp LLC), while all analyses were performed in R version 4.4.1 or higher (R Core Team, 2024). Packages *haven*, *magrittr*, and *dplyr* were used for data management, while packages *survival* and *survminer* were used for survival analysis. The *MatchIt* package (version 4.5.5 or higher) was used to implement PSM.[5]

Supplementary Table 1. Availability and definition of data elements in the HOVON and RWD cohorts

**Parameter**	**Data source** *HOVON*	*RWD*
Patient characteristics		
Age at diagnosis	✓	✓
Sex	✓	✓
WHO performance status at diagnosis	✓	±
Comorbidities[a]	✓	✓
Inclusion in previous or concurrent clinical trials	✓	✓
Disease characteristics		
White blood cell count at diagnosis	✓	✓
Cytogenetics[b]	✓	±
Molecular data[b]	✓	±
ELN 2017 AML risk classification[b]	✓	±
Treatment regimen	✓	✓
Outcome		
CR rate	✓	±[c]
AlloSCT	✓	✓
Relapse	✓	✗[d]
OS	✓	✓
EFS	✓	✗[d]
Follow-up monitoring	✓	✓
Adverse events		
Toxicity monitoring	✓	✓[e]

[a] Comorbidities were registered according to the Hematopoietic cell transplantation (HCT)-specific comorbidity index; not all comorbidities could be registered in the RWD cohort, as for example, pulmonary function tests were not frequently performed in routine diagnostics outside the context of a clinical trial

[b] Performed based on ELN 2017 guidelines in the HOVON-155 trial, and therefore also evaluated as per ELN 2017 in the RWD cohort; however, some ELN aberrations, such as t(6;9)(p22.3;q34.1) and t(v;11), were not recorded in the RWD cohort

[c] Treatment response is registered for some chemotherapy cycles in the Netherlands Cancer Registry; the prospective monitoring system has improved on the reporting of response data, but altogether the data are not as comprehensive as in the HOVON-155 trial

[d] Relapse was not routinely registered in the NCR database and is currently not yet available from automated extraction by the Datagateway; this also precluded the use of EFS as an endpoint of interest

[e] While not all adverse events of the HOVON-155 trial were collected in the RWD cohort, the ones that were deemed clinically most relevant were derived according to CTCAE (grade ≥ 3) guidelines

Abbreviations: AlloSCT: Allogeneic stem cell transplant; CR: complete remission; EFS: event-free survival; ELN: European Leukemia Net; OS: overall survival; RWD: Real-World Data; WHO: World Health Organization

Supplementary Table 2. Assessment of the matching procedures

**Matching method**	**Matched cohorts**	
	H155 control arm patients vs. RWD	
	Std. Mean Diff.	Var. Ratio
Unmatched		
Propensity score	1.31	0.97
Age at diagnosis	-0.33	0.55
ELN 2017 risk classification		
Favorable	0.18	-
Intermediate	0.50	-
Adverse	0.48	-
Unknown	-1.40	-
HCT-CI		
0	-0.19	-
1-2	-0.21	-
3+	0.33	-
1:1 NN caliper PSM		
Propensity score	0.12	1.12
Age at diagnosis	-0.15	0.44
ELN 2017 risk classification		
Favorable	0.08	-
Intermediate	0.04	-
Adverse	-0.04	-
Unknown	-0.05	-
HCT-CI		
0	0.05	-
1-2	-0.04	-
3+	0.00	-
Coarsened exact matching		
Age at diagnosis	0.12	0.66
ELN 2017 risk classification		
Favorable	0.00	-
Intermediate	0.00	-
Adverse	0.00	-
Unknown	0.00	-
HCT-CI		
0	0.00	-
1-2	0.00	-
3+	0.00	-
Exact matching followed by caliper PSM		
Propensity score	0.06	1.05
Age at diagnosis	-0.24	0.80
ELN 2017 risk classification		
Favorable	0.00	-
Intermediate	0.00	-
Adverse	0.00	-
Unknown	0.00	-
HCT-CI		
0	0.00	-
1-2	0.00	-
3+	0.00	-

Abbreviations: HCT-CI: Hematopoietic cell transplantation (HCT)-specific comorbidity index; NN: Nearest Neighbor; PSM: Propensity Score Matching; RWD: Real-World Data; Std. Mean Diff.: Standardized Mean Difference; Var. Ratio: Variance Ratio

Supplementary Table 3. Survival outcome of H155 control arm patients and RWD patients under various matching algorithms

	H155 control arm patients vs. RWD			
Outcome	ESS	2-year estimate±SE	HR (95% CI)	P
OS				
1:1 NN caliper PSM	51 vs. 51	18±5 vs. 18±5	1.05 (0.69-1.62)	0.81
CEM	52 vs. 20.68	23±6 vs. 15±6	1.07 (0.68-1.69)	0.76
EM + caliper PSM	47 vs. 47	23±6 vs. 17±6	0.91 (0.62-1.34)	0.63
IPTW	70 vs. 120	23±5 vs. 20±6	0.90 (0.56-1.47)	0.68

Abbreviations: CEM: coarsened exact matching; CI: confidence interval; EM: exact matching; ESS: effective sample size; HR: hazard ratio; IPTW: inverse probability of treatment weighting; OS: overall survival; PSM: propensity score matching; RWD: Real-World Data; SE: standard error

Supplementary Table 4. Reported toxicity (CTCAE grade ≥3) of H155 control arm patients and RWD patients in the unmatched cohorts

Parameter	**H155 control arm** (n = 70)	**RWD** (n = 120)
Acute renal insufficiency grade ≥3, n (%)	2 (2.9)	10 (8.3)
Bleeding grade ≥3, n (%)	10 (14.3)	8 (6.7)
Intensive care unit admission, n (%)	-	1 (0.8)
Hepatic toxicity grade ≥3, n (%)	5 (7.1)	7 (5.8)
Bacteremia, n (%)	6 (8.6)	5 (4.2)
Venous thrombosis grade ≥3, n (%)	2 (2.9)	2 (1.7)
Pulmonary aspergillosis, n (%)	7 (10.0)	10 (8.3)

Abbreviations: CTCAE: Common Terminology Criteria for Adverse Events; RWD: Real-World Data

Supplementary Figure 1. CONSORT diagram for inclusion of H155 control arm patients and Real-World Data (RWD) patients

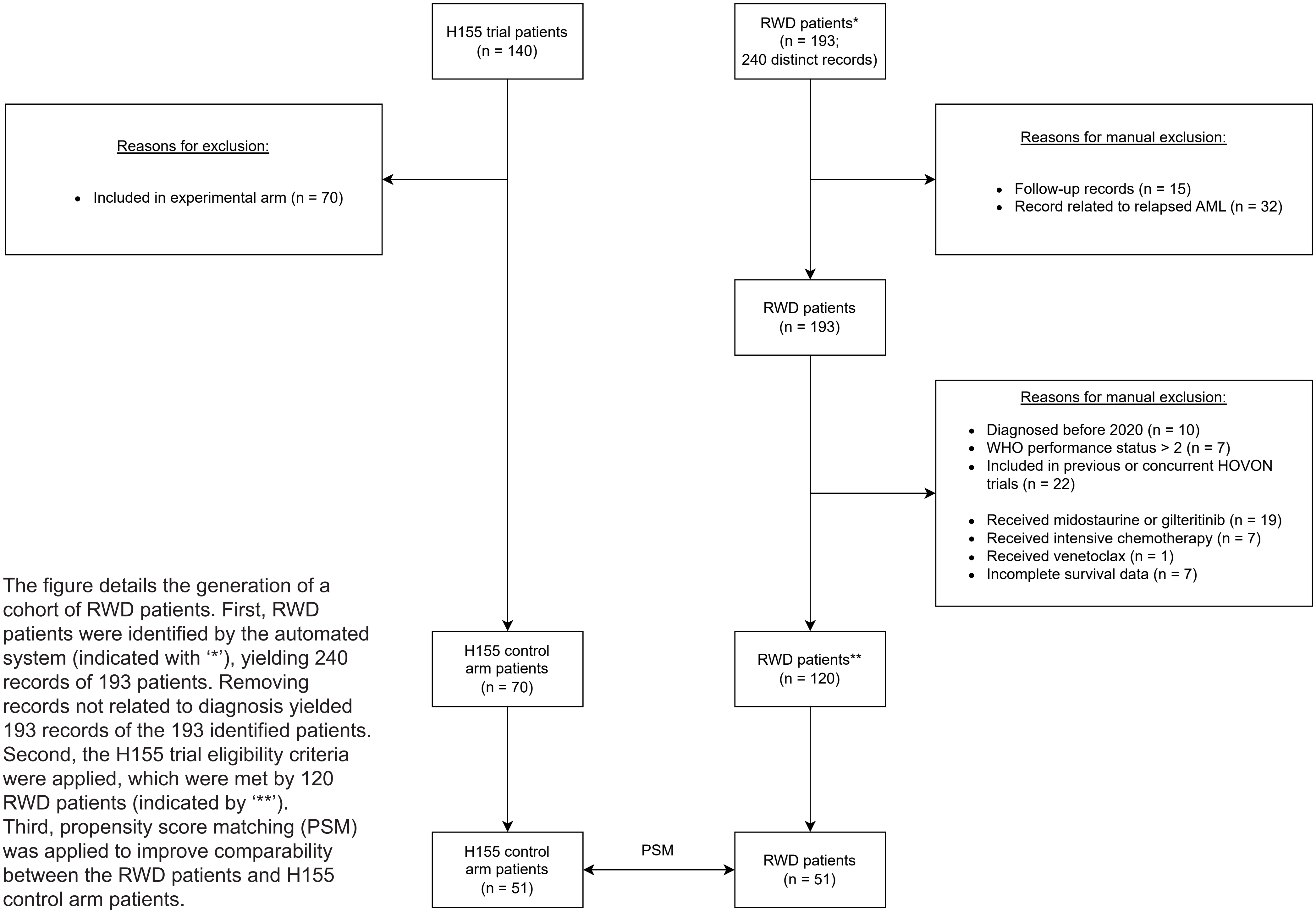

The figure details the generation of a cohort of RWD patients. First, RWD patients were identified by the automated system (indicated with ‘*’), yielding 240 records of 193 patients. Removing records not related to diagnosis yielded 193 records of the 193 identified patients. Second, the H155 trial eligibility criteria were applied, which were met by 120 RWD patients (indicated by ‘**’). Third, propensity score matching (PSM) was applied to improve comparability between the RWD patients and H155 control arm patients.

Supplementary Figure 2. Distribution of propensity scores for H155 control arm patients and RWD patients in the unmatched and matched cohorts

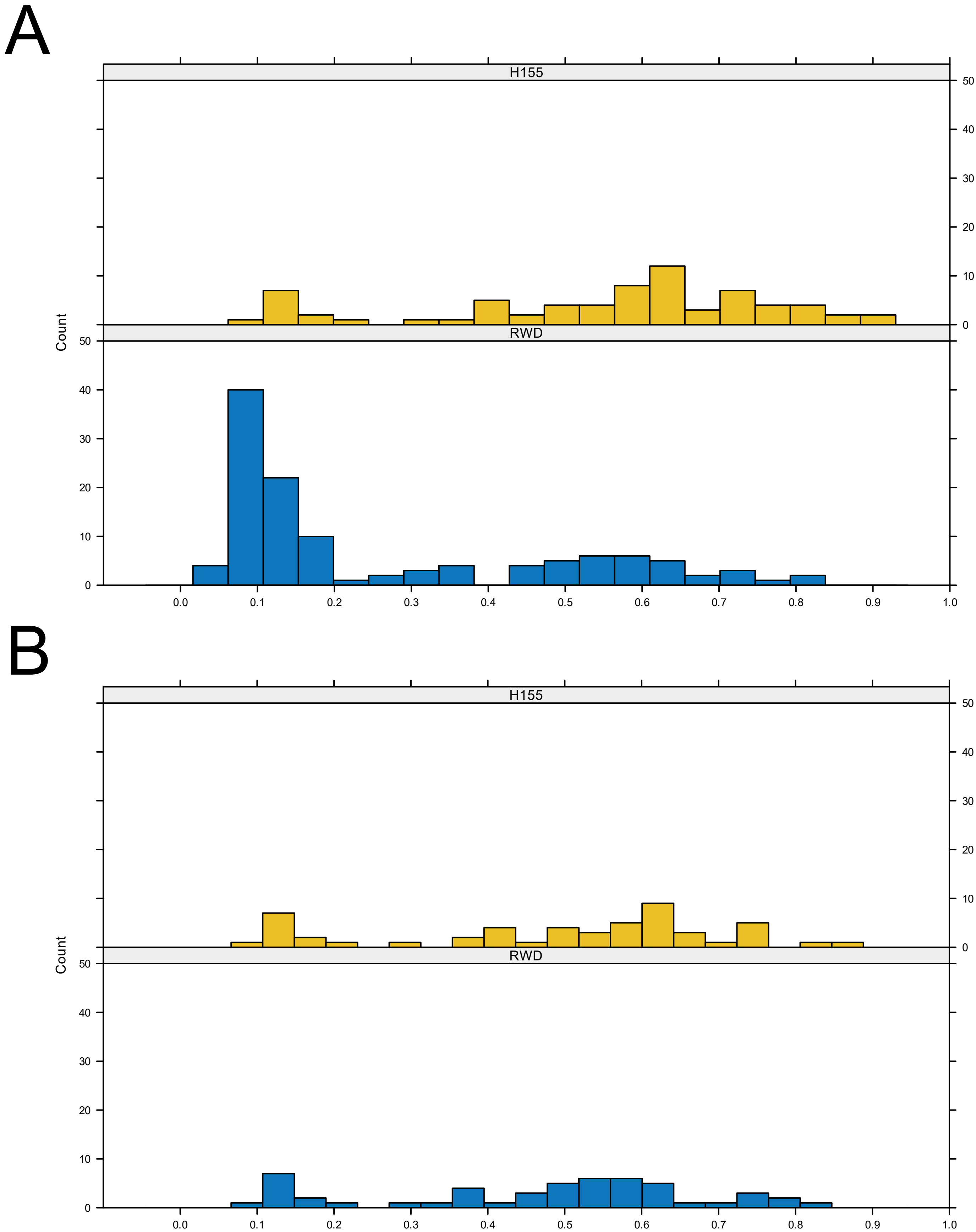

The histograms detail the distribution of the propensity scores in the H155 control arm patients and RWD cohort prior to and after matching. Cohorts were matched 1:1 using caliper NN propensity score matching for age, ELN2017 risk classification, and comorbidities as determined by the HCT-CI. Panel A: histograms detailing the distribution of propensity scores before matching. Panel B: histograms detailing the distribution of propensity scores after matching. Abbreviations: HCT-CI: Hematopoietic cell transplantation (HCT)-specific comorbidity index; NN: nearest neighbor; RWD: Real-World Data

References

1. Huls G, Chitu DA, Tick L, Boersma R, Breems D, Herbers A, et al. Midostaurin added to 10-day decitabine, for patients unfit for intensive chemotherapy with AML and higher risk MDS, irrespective of FLT3 mutational status, does not improve outcome. Ann Hematol. 2025 Jan;104(1):361–8.

2. Dinmohamed AG, Visser O, van Norden Y, Huijgens PC, Sonneveld P, van de Loosdrecht AA, et al. Trends in incidence, initial treatment and survival of myelodysplastic syndromes: a population-based study of 5144 patients diagnosed in the Netherlands from 2001 to 2010. Eur J Cancer. 2014 Mar;50(5):1004–12.

3. Langhout SAM, Hermans SJF, Smit AJT, Berkx E, Kurk SA, Schade KJ, et al. Real-time data in cancer registries: Validation of an automated data extraction system. iScience. 2025 Jul;113056.

4. Döhner H, Estey E, Grimwade D, Amadori S, Appelbaum FR, Büchner T, et al. Diagnosis and management of AML in adults: 2017 ELN recommendations from an international expert panel. Blood. 2017 Jan 26;129(4):424–47.

5. Ho DE, Imai K, King G, Stuart EA. MatchIt : Nonparametric Preprocessing for Parametric Causal Inference. J Stat Softw [Internet]. 2011 [cited 2025 May 6];42(8).

6. VanderWeele TJ, Ding P. Sensitivity Analysis in Observational Research: Introducing the E-Value. Ann Intern Med. 2017 Aug 15;167(4):268.
